# Non-Invasive Assessment of the Intraventricular Pressure Using Novel Color M-Mode Echocardiography in Animal Studies: Current Status and Future Perspectives in Veterinary Medicine

**DOI:** 10.3390/ani13152452

**Published:** 2023-07-29

**Authors:** Ahmed S. Mandour, Ahmed Farag, Mahmoud A. Y. Helal, Gamal El-Masry, Salim Al-Rejaie, Ken Takahashi, Tomohiko Yoshida, Lina Hamabe, Ryou Tanaka

**Affiliations:** 1 Department of Animal Medicine (Internal Medicine), Faculty of Veterinary Medicine, Suez Canal University, Ismailia 41522, Egypt; 2 Veterinary Surgery, Tokyo University of Agriculture and Technology, Tokyo 183-0054, Japan; 3 Department of Surgery, Anesthesiology, and Radiology, Faculty of Veterinary Medicine, Zagazig University, Zagazig 44519, Egypt; 4 Animal Medicine Department, Faculty of Veterinary Medicine, Benha University, Moshtohor, Benha 13736, Egypt; 5 Agricultural Engineering Department, Faculty of Agriculture, Suez Canal University, Ismailia 41522, Egypt; 6 Department of Pharmacology & Toxicology, College of Pharmacy, King Saud University, Riyadh 11564, Saudi Arabia; 7 Department of Pediatrics and Adolescent Medicine, Juntendo University Graduate School of Medicine, Bunkyo, Tokyo 113-8421, Japan; 8 Department of Veterinary Surgery, Division of Veterinary Research, Obihiro University of Agriculture and Veterinary Medicine, Hokkaido 080-8555, Japan

**Keywords:** color M-mode echocardiography, intraventricular pressure gradients, diastolic function, echocardiography, heart failure, animal models

## Abstract

**Simple Summary:**

Traditional echocardiographic imaging mainly identifies cardiac diseases when symptoms are clear. This makes the innovation of new methods for the prediction and early diagnosis of cardiac diseases inevitable, especially when diastolic function evaluation is needed. Generally, non-invasive assessment of heart function is currently attracting great attention away from sophisticated techniques. With the development of color M-mode echocardiography (CMME), the derived intraventricular pressure differences (IVPDs) and intraventricular pressure gradients (IVPGs) created a breakthrough in the non-invasive assessment of diastolic function in animal models as well as in some companion animals and human studies. They are beneficial for continuous data acquisition without invasive interference, and their results showed good relatedness with the measurements acquired from the catheterization technique.

**Abstract:**

The assessment of diastolic function has received great interest in order to comprehend its crucial role in the pathophysiology of heart failure and for the early identification of cardiac events. Silent changes in the intraventricular flow (IVF) dynamics occur before the deterioration of the cardiac wall, although they cannot be detected using conventional echocardiography. Collective information on left ventricular (LV) pressures throughout the cardiac cycle has great value when dealing with patients with altered hemodynamics. Accurate pressure measurement inside the ventricle can be obtained by invasive methods to determine the LV diastolic pressures, which reflect the myocardial relaxation and compliance. However, catheterization is only feasible in the laboratory setting and is not suitable for clinical use due to its disadvantages. In contrast, echocardiography is simple, safe, and accessible. Color M-mode echocardiography (CMME) is an advanced cardiac evaluation technique that can measure the intraventricular pressure differences (IVPDs) and intraventricular pressure gradients (IVPGs) based on the Doppler shift of the IVF. Recently, the assessment of IVPD and IVPG has gained growing interest in the cardiovascular literature in both animal and human studies as a non-invasive method for the early diagnosis of cardiac dysfunctions, especially diastolic ones. The usability of IVPD and IVPG has been reported in various surgically induced heart failure or pharmacologically altered cardiac functions in rats, dogs, cats, and goats. This report aims to give an overview of the current studies of CMME-derived IVPD and IVPG in animal studies and its feasibility for clinical application in veterinary practice and to provide the prospects of the technique’s ability to improve our understanding.

## 1. Introduction

Heart failure is one of the most common causes of death globally; these cases include both diastolic and systolic dysfunctions. In humans and companion animals, heart failure is the second leading cause of death after cancer [1,2]. The development of novel echocardiographic techniques that help in the early detection of cardiac diseases is a major objective of cardiologists [3]. Echocardiography is the “basic method of cardiac examination”, and it has become the first choice for structural and functional evaluation of the heart, not only in humans and companion animals, but also in horses and, in general, in all animals [4,5]. Although multiple diagnostic laboratory markers and imaging approaches can accurately detect cardiac insufficiency, most of the established techniques can only diagnose cardiac abnormalities when the clinical symptoms of heart failure become overt. Therefore, minimizing the worldwide increase in cardiovascular diseases necessitates the establishment of novel diagnostic approaches that help in the early detection of cardiac diseases.

Currently, it is believed that measuring diastolic function is crucial for a thorough understanding and early identification of heart diseases since diastolic dysfunction (DD) precedes systolic dysfunction in many cardiac disorders [6,7]. Different clinical states, such as restrictive cardiomyopathy, infiltrative myocardial disease, and, in particular, diastolic failure (also known as heart failure with preserved systolic function), which is present in a significant number of patients with pulmonary congestion, are all caused by an abnormal diastolic function of the left ventricle (LV) [8]. Clinical evaluation of the diastolic function using traditional echocardiographic methods cannot accurately detect diastolic dysfunction, as it is mainly related to heart rate, loading states, and arrhythmias. To prevent overestimation, the echocardiographer must adopt a methodical approach to the investigation of LV diastolic function. This includes the incorporation of Doppler patterns with additional echocardiographic parameters such as chamber dimensions, wall thicknesses, systolic function, valve function and morphology, and clinical data [9,10].

Despite numerous methods that have been established to determine diastolic performance, the evaluation of diastolic parameters by catheterization remains the gold standard method which can efficiently measure compliance, relaxation of the myocardium, and filling pressure. However, its invasiveness and the requirement of preoperative measures limit its clinical applicability [11,12]. Likewise, magnetic resonance imaging (MRI) and computed tomography (CT) have limited accessibility in veterinary clinics because of high costs, time consumption, and the requirement of anesthesia. In addition, catheterization, CT, and MRI methods are difficult to repeat on the same individual, while repeatability is necessary for these methods when serial data collection is needed, such as in chronic cardiac disease modalities.

Recently, with the development of color M-mode echocardiography (CMME), the derived intraventricular pressure differences (IVPDs) and intraventricular pressure gradients (IVPGs) have created a breakthrough in the non-invasive assessment of diastolic function in animal models as well as in some companion animals and human studies. A literature search was conducted in English through PubMed databases and the publications included in this review are up to January 2023. Selected articles were chosen based on the presence of animal studies having either experimental, preclinical, or clinical trials using the following keywords: color M-mode echocardiography, intraventricular pressure gradients, IVPG, intraventricular pressure difference, IVPD, diastolic function.

## 2. CMME-Derived IVPG/IVPD for Assessment of Cardiac Function

Generally, it is widely believed that silent changes in the IVF dynamics occur earlier than deteriorations of the cardiac wall; however, IVF changes cannot be detected by conventional echocardiography. The development of Doppler echocardiography has allowed non-invasive measurement of the intracardiac flow. In the normal heart, there is an IVF dynamic including pressure differences. The IVPD is generally defined as the pressure difference that is generated in early diastole between distinct segments within the LV when the apical pressure in the LV drops below the basal pressure [13]. Pressure disparity within the LV, which is obtained principally by catheterization, is responsible for the LV suction force that withdraws the blood from the left atrium (LA) to the inside of the LV. The blood flow propagation inside the LV is considered when developing the IVPD as measured by the catheterization method. The physiological differences in the LV pressure are a healthy phenomenon that occurs in the normal heart during both phases of LV activity: during LV filling in early diastole to promote the suction force, and during ventricular systole to enhance the contractility force [14].

Assessment of IVPD was first uncovered in 1979 when Ling et al. [15] reported for the first time the IVPD between the LV base and apex during early diastole. After that, Falsetti et al. [16] explained the possible clinical importance of intraventricular pressure stratification. They observed a gradual increase in the magnitude of IVPD during isoproterenol infusions in a graded manner and a reduction in IVPD with propranolol administration. At this time, an assessment of IVPD was performed by a conductance catheter method.

The CMME-derived IVPD measurements have started to be used as a non-invasive metric for cardiac function evaluation because of the rapid advancement of echocardiographic technology and the introduction of CMME. Since the IVPD has been approximated from CMME by solving Euler equations, it became possible to determine the sucking force of the ventricle without using any intrusive methods, as stated by Greenberg et al. [17]. Since then, various animal studies have been conducted to uncover the pathophysiologic role of IVPD and IVPG in the development of cardiac failure to highlight the potential role of these indices in the diagnosis of heart failure.

## 3. CMME Scanning and the IVPD and IVPG Calculation

Takahashi and colleagues [18] modified the CMME method, which was previously used in adult humans [19,20] to measure IVPG in young children. Later, the same method was used on rats, dogs, and cats [21,22,23,24,25]. This method assumes dividing the LV into three segments (basal, mid, and apical) in the longitudinal axis, and the respective IVPD and IVPG can be calculated. For evaluation of the cardiac function by CMME, it is important to be aware of proper machine settings, trace a constant LV inflow tract, and understand the basis of IVPD calculation and software analysis procedures. The left apical four-chamber or two-chamber view should be maintained consistently by 2D echocardiography to enable visualization of the entire inflow tract from the LA to the apex of the LV without interfering with the outflow tract to avoid erroneous imaging [26].

**Figure 1:**
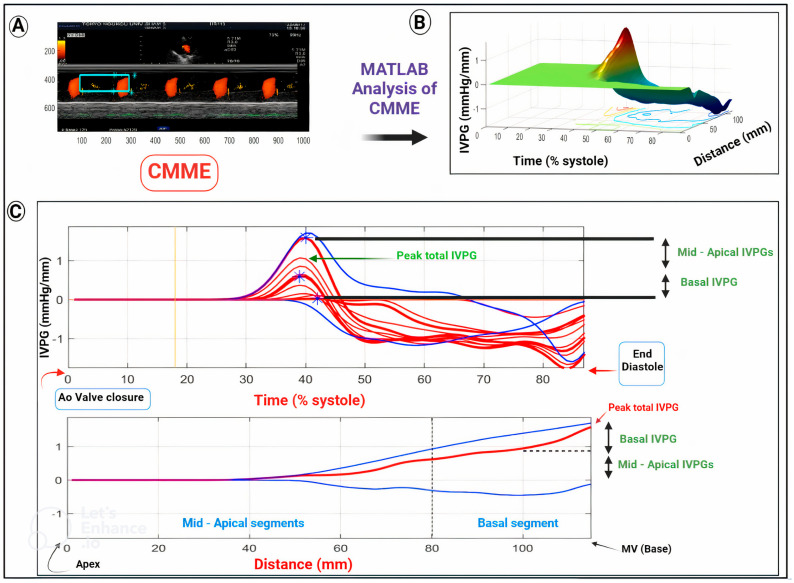
Schematic illustration of IVPG calculation after examination of the LV via color M-mode echocardiography (CMME). The same steps are used regardless of the species. After optimization of the LV inflow from the left apical view by color flow Doppler, IVPG is switched on to trace the entire scanline of the LV inflow tract, from the LV base (mitral valve, MV) to the apex (**A**). Then, captured CMME images are used for IVPG measurements in the region of interest (ROI, blue box) using MATLAB software (**B**,**C**). After solving Euler’s equation, 3-dimensional IVPG (**B**) with a *Z*-axis, which represents the distance (mm) from the apex of the LV to its base (mitral valve), and the temporal and spatial profiles (**C**) of the novel indices are automatically calculated. Results of IVPG are obtained over a percent of the time of systolic duration which elapsed from the complete closure of the aortic valve (Ao) to the end of the diastole. Calculated IVPD or IVPG are displayed as a two-dimensional graph of the temporal and spatial profiles in which both peak and segmental IVPD or IVPG are presented (**C**). The first curve represents the IVPG from the closure of the aortic valve to the end of the diastolic phase, while the second one represents the IVPG along the entire left ventricle, from the base to the apex. The red line represents the spatial profile at peak total IVPG that occurs at the left ventricular base. The upper and lower blue lines represent the inertial and the convective IVPG, respectively (**C**). In all calculations, the same curves can represent IVPD or IVPG (IVPG is obtained when IVPD is divided by the left ventricular length).

MATLAB (MATLAB software, Version 2019a, The MathWorks, Natick, MA, USA) is used to calculate the IVPD following CMME image acquisition (Figure 1). The following Euler equation is used to compute the IVPD of each related portion:

(∂P)/(∂s) = −ρ((∂v)/(∂t) + v (∂v)/(∂s)),(1)

where ∂ denotes an elemental change, P denotes pressure, ρ denotes constant blood density (1060 kg/m^3^), v denotes velocity, s denotes position along the color M-mode streamline, and t denotes time. MATLAB is used to obtain v, s, and t and then measure the relative pressures in the region of interest [17,21,27]. The IVPD and IVPG can be determined by solving the Euler equation assuming laminar blood flow across the mitral valve and the ultrasound scanline is connected to the inflow streamline [28].

*IVPG* is calculated by dividing the *IVPD* by the *LV* length to exclude the effect of the left ventricular size using the following equation [29]:

(2) $$IVPG\;\left(mmHg/cm\right)=\frac{IVPD}{LVlength}$$

## 4. Usability of IVPD and IVPG in Heart Failure

A. ***Rat models of cardiovascular disorders***

**Figure 2:**
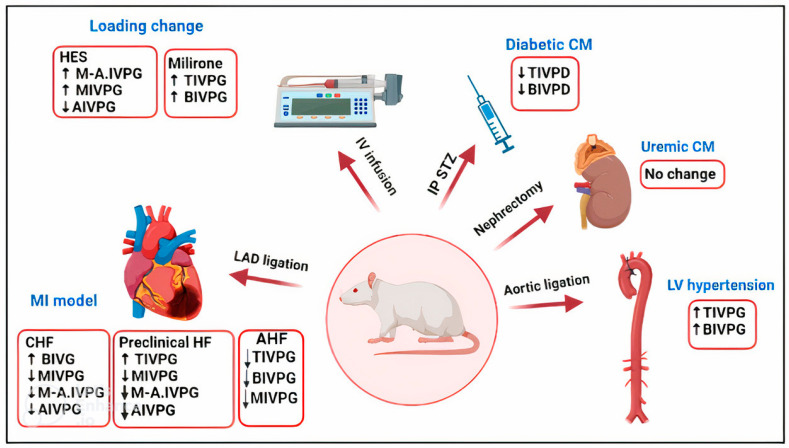
Schematic illustration of novel intraventricular pressure difference (IVPD) and intraventricular pressure gradient (IVPG) variables derived from Color M-mode echocardiography (CMME) in rat experiments. HES, hydroxyethyl starch, CHF, congestive heart failure; LV, left ventricle; CM, cardiomyopathy; STZ, streptozotocin; IP, intraperitoneal; IV, intravenous; LDA, left descending artery. TIVPG, total IVPG; BIVPG, basal IVPG; MIVPG, mid-IVPG; M-A.IVPG, mid-to-apical IVPG. TIVPD, total IVPD; BIVPD, basal IVPD.

Updated studies that used CMME-calculated IVPD and IVPG measurements in heart failure are illustrated in murine studies (Figure 2 and Table 1).

(a) ***Changes in IVPD and IVPG in loading conditions***

The current conventional echocardiographic evaluation methods for the assessment of diastolic function have certain recognized limitations, especially under various loading states. The accuracy of conventional echocardiographic methods for diagnosis of DD, such as mitral inflow E-wave velocity (E) using pulsed-wave Doppler echocardiography and mitral annular early diastolic velocity (Em) using tissue Doppler imaging (TDI), is controversial, since they can be influenced by loading conditions with limited efficiency in expressing regional myocardial dysfunction [6].

The effect of loading changes on IVPD and IVPG variables measured through CMME was studied in rats after hydroxyethyl starch and milrinone infusion. The result revealed that mid-to-apical IVPG, mid-IVPG, and apical IVPD were elevated significantly with milrinone infusion; meanwhile, total IVPD and basal IVPD were significantly increased following the infusion of hydroxyethyl starch [22]. The abovementioned results suggested that IVPD and IVPG could be useful indices to evaluate various loading conditions in rats, especially when continuous data acquisition is required, as in the chronic cardiac disease model (Figure 2, Table 1).

(b) ***Hypertrophic cardiomyopathy (HCM)***

Advanced heart failure secondary to HCM frequently occurs in humans and cats. Nonetheless, there is still a limited clinical ability of current diagnostic approaches to detect HCM in patients at risk of heart failure [30,31]. DD is common in HCM, which occurs due to the dissociation of the active component of actin–myosin in the early filling phase and the passive compliance of the LV. DD reduces the rate and magnitude of LV filling and reduces the SV. This results in the elevation of left ventricular end-diastolic pressure (LVEDP), leading to symptoms of heart failure [32].

During the development of LV hypertrophy in the rat model, total and basal IVPG was increased approximately one month after aortic coarctation (Figure 2, Table 1). Moreover, treatment of these rats with salvianolic acid B and carvedilol resulted in reduced basal IVPG, suggesting that salvianolic acid B and carvedilol may improve cardiac function during the development of LV hypertrophy through reverse reduction of the elevated IVPG [24].

In veterinary practice, feline HCM is the most frequently detected heart disease in cats; it occurs due to breed predisposition (cat breeds include Persian, Maine Coon, Ragdoll, British Shorthair, Sphynx, and Chartreux) or due to several genetic mutations. However, for most cats with HCM, the cause is unknown [33,34]. Enlarged LA, congestive heart failure (CHF), and thromboembolism are related to decreased survival in cats with HCM. In addition, LA function, LV hypertrophy, and LV systolic function evaluation by conventional echocardiography are used to evaluate the prognosis [35]. These variables are associated with increased deaths in cats with HCM; however, deaths also occur in asymptomatic cats [36]. The prediction of heart failure in cats with HCM is still controversial and requires intensive multidisciplinary studies [31,37]. Until now, CMME-derived IVPG has not been investigated in feline HCM. A quantitative assessment of IVPD and IVPG in cats via CMME has been recently published [25]. Therefore, detailed investigations are still needed to characterize the changes in IVPD and IVPG via CMME in cats with preclinical and symptomatic HCM and to examine the response of the heart of the diseased cats to the medication.

(c) ***Diabetic cardiomyopathy***

The number of human patients with diabetes mellitus (DM) is rapidly growing in the industrialized world. In DM patients, subclinical LV cardiomyopathy and LA dysfunction usually occur [38]. The pathophysiology of DM is complex and the cardiovascular consequences are still not fully uncovered despite the long-time discovery of the relationship [39]. Diabetic heart failure is associated with histomorphological alterations, formation of nonenzymatic advanced glycation end products, reduced cardiac compliance, and myocardial ischemia. Hypertension also occurs in DM with different underlying mechanisms, including increased sympathetic tone, renin–angiotensin–aldosterone system (RAAS) activation, irregularities in insulin-mediated vasodilation, artery damage, and aberrant immunological and inflammatory responses [40]. The prognosis for patients with DM who have subclinical cardiac dysfunction is improved by early identification and initiation of therapies that can prevent or reverse heart failure [41]. Cardiomyopathy secondary to DM is a common finding in diabetic patients. Until now, traditional 2D echocardiography, which is commonly used in cardiology practice, is incapable of the characterization or early diagnosis of uncomplicated reduction in myocardial function, since 2D echocardiography is unable to estimate LV longitudinal function [42].

Characterization of cardiac dysfunction in DM patients usually appears in the advanced stages of the disease. A recent trial found a reduction in longitudinal deformation in all layers and epicardial and mid-myocardial circumferential deformation at the basal level in type-1 DM patients using speckle-tracking echocardiography (STE) [43]. Another study showed increased LA stiffness and decreased LA phasic function in patients with DM type-1 [44].

In an experimental trial, Kitpipatkun et al. studied the pathophysiologic changes in the IVF in diabetic cardiomyopathic rats, which was experimentally induced via streptozotocin. In this study, diastolic failure and delayed cardiac relaxation were observed in diabetic rats, as indicated by reduced mitral E velocity, E/A ratio, TDI measurements, and mid-to-apical IVPD [23].

(d) ***Myocardial infarction model***

Myocardial infarction (MI) is primarily a type of ischemic heart disease that is caused by an extended shortage of oxygen to the cardiac tissues, which occurs due to constriction or blockage of one or more branches of the coronary circulation [45,46]. The development and build-up of fibrotic scars in MI patients over time will harm the cardiac structure and function [47].

Two studies have been conducted to evaluate the usefulness of IVPG derived from CMME in MI models in rats (Figure 2, Table 1). The first study was conducted on a chronic MI model in rats that developed heart failure lasting 6 months [48,49]. In this study, CMME-derived IVPG segments were important for the cluster classification of rats based on the existence of heart failure. Moreover, CHF rats showed significantly increased segmental IVPG, which was significantly correlated with the hemodynamic findings measured from the intracardiac catheter, suggesting the ability of IVPG to predict the CHF progression caused by MI [49].

The second study was conducted to evaluate the efficiency of IVPG to detect subclinical heart failure in rats which were maintained for one month after the induction of MI, as well as to evaluate the efficiency of the new cardioprotective medication, trehalose, on cardiac function [50]. In the later study, Farag et al. found that segmental IVPG was significantly reduced in MI rats with subclinical heart failure, which was slightly improved after trehalose treatment. However, our findings in the aforementioned study offer a valuable correlation between IVPG, heart rate variability parameters, and other conventional echocardiographic variables in the investigated groups.

(e) ***Uremic cardiomyopathy***

In individuals with renal failure, uremic cardiomyopathy (UC) is traditionally characterized by diastolic dysfunction in conjunction with left ventricular hypertrophy and myocardial fibrosis. [51,52]. LV hypertrophy in UC patients generally originates from complex pathophysiological consequences, including pressure overload due to systemic hypertension causing concentric hypertrophy, volume overload due to increased blood volume, anemia causing eccentric hypertrophy, and the uremic state itself [53,54]. In the rat model of UC induced by partial nephrectomy, there was no significant difference in the IVPG variables between rats with UC and sham rats, suggesting that myocardial dysfunction occurs earlier than the IVF change in this disease [55].

**Table 1:** Color M-mode-derived IVPD and IVPG measurements in rat models of heart failure.

Model	Left Ventricular Hypertrophy			Uremic CM		Diabetic CM	Loading Change		Infarction for 6 Months		Infarction for 1 Month	
Groups	LVH	Carvedilol	Sal-B	UCM	Sal-B	DM	MIL	HES	+ve CHF	−ve CHF	MI	MI + Tre^®^
Induction	Aortic coarctation			kidney dissection		Streptozotocin	IV infusion		LAD ligation			
Total IVPG	↑	↓	↓	NS	NS	ND	NS	↑	NS	NS	↓	NS
Basal IVPG	↑	↓	↓	NS	NS	ND	NS	↑	↑	↑	↓	NS
Mid-to-apical IVPG	NS	NS	NS	NS	NS	ND	↑	NS	↓	↓	NS	NS
Mid IVPG	ND	ND	ND	ND	ND	ND	↑	NS	↓	↓	↓	↓
AIVPG	ND	ND	ND	ND	ND	ND	↑	NS	↓	↓	NS	NS
Total IVPD	ND	ND	ND	ND	ND	↓	NS	↑	ND	ND	ND	ND
Basal IVPD	ND	ND	ND	ND	ND	↓	↓	↑	ND	ND	ND	ND
Mid IVPD	ND	ND	ND	ND	ND	NS	↑	NS	ND	ND	ND	ND
Mid-to-apical IVPD	ND	ND	ND	ND	ND	ND	↑	NS	ND	ND	ND	ND
Apical IVPD	ND	ND	ND	ND	ND	NS	↑	↑	ND	ND	ND	ND
Reference	[24]			[55]		[23]	[22]		[48,49]		[50]	

Collective studies on IVPD (intraventricular pressure difference) and/or IVPG (intraventricular pressure gradient) in rat models of heart failure. LVH, left ventricular hypertrophy; CM, cardiomyopathy; DM, diabetes mellitus; Sal-B, Salvianolic acid B; HES; hydroxyethyl starch; MI +ve CHF, myocardial infarction developed congestive heart failure; MI −ve CHF, myocardial infarction without congestive heart failure; Tre^®^, trehalose; NS, not significant; ND, not detected. ↑, significant increase; ↓, significant decrease.

B. ***IVPD and IVPG in experimental studies in dogs***

**Figure 3:**
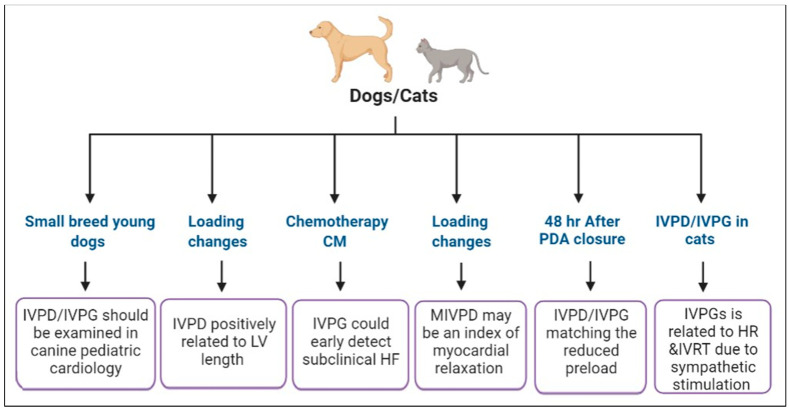
Changes in IVPD and IVPG calculated from color M-mode echocardiography in experimental and clinical studies in companion animals. CM, cardiomyopathy; PDA, patent ductus arteriosus; HF, heart failure; MIVPD, mid IVPD.

Figure 3 and Table 2 show the IVPD and IVPG measurements via CMME analysis in dog studies.

(a) ***Changes in IVPD and IVPG in loading conditions***

Matsuura et al. experimentally sought the IVPD changes under different loading conditions in dogs using transthoracic CMME echocardiography and pressure–volume conductance catheter (Figure 3, Table 2). In the latter study, overloading models were achieved by alteration of the afterload through balloon dilatation of the thoracic aorta, preload change via hydroxyethyl starch infusion, and after milrinone medication to alter the LV relaxation. They found that mid-IVPD decreased under pressure loading and increased during milrinone administration, but it did not change under volume loading [56].

(b) ***Chemotherapy-induced heart failure***

Because DD can occur before the systolic failure, it may be possible to identify diastolic ventricular dysfunction preclinically. In adult cardiac disorders, IVPG is considered an important index of myocardial diastolic function [6,9,57]. Cardiotoxicity due to chemotherapy is one of the most common limitations of the treatment of cancers [58,59]. Anthracycline is a famous drug family that shows cardiotoxicity side effects in both animals and humans [60,61]. Characterization of early cardiac dysfunction in patients receiving chemotherapy may allow early intervention and subsequently reduce the risk of heart failure in cancer patients [26]. Research trials speculated on the utility and efficiency of non-invasive IVPG from CMME as a sensitive marker for the early identification of chemotherapy-associated cardiac dysfunction after anticancer drug administration [62].

The utility of IVPG was examined for the early identification of subclinical LV dysfunction caused by doxorubicin in Beagle dogs (Figure 3, Table 2). The study provided a long-term investigation of heart function using a combination of conventional echocardiography, cardiac catheterization (measure stiffness constant β, Emax, Tau, LVEDP), CMME-derived IVPG, and STE (measure longitudinal strain, strain rate, and LV twist) [26]. The results revealed that conventional cardiac parameters and STE fail to identify the subclinical cardiomyopathy, whereas the total IVPG and mid-IVPG were significantly reduced after the end of the treatment protocol (four months) and continued for 1.5 years. Additionally, in the same study, mid-IVPG was found to be a good predictor of Emax [26]. According to this study, the first change noticed with doxorubicin cardiotoxicity in dogs was impaired contractility by catheterization, and the only non-invasive method that could detect subclinical changes in cardiac function was IVPG. Furthermore, it has been shown that the total and mid-to-apical IVPG may represent a novel marker to identify early changes in cardiac function after anthracycline therapy in children [62]. Therefore, IVPG derived from CMME may be a potential diagnostic index in patients treated with chemotherapy, which deserves further clinical studies.

C. ***IVPD and IVPG measurements in the clinical setting in companion animal medicine***

IVPD and IVPG indices were evaluated in a serial study in dogs with pharmacologically modulated cardiac function as well as normal healthy dogs. Updated studies that used CMME-calculated IVPD and IVPG measurements in companion animals are illustrated in Figure 3. Until now, the feasibility of IVPD and/or IVPG measured by CMME in healthy companion animals has been published [21,25,63]. Firstly, Matsuura et al. reported the IVPD and IVPG measurements in 50 different client-owned dogs [21]. They found a positive correlation between IVPD and LV length; meanwhile, IVPG did not show a significant correlation. This result revealed that when utilizing IVPD in the field of veterinary medicine, LV size should be considered, whereas IVPG can be utilized when comparing groups of different heart sizes. Secondly, IVPG was increased by the HR and short isovolumic relaxation time in cats, which was seen as a kind of physiological adaptation to the adrenergic reaction, which seems to be linked to a rise in the sympathetic tone during the handling of cats [25]. Thirdly, IVPD and IVPG were also examined in a group of young breed dogs as an initial step to include the non-invasive assessment of IVPD and IVPG in veterinary pediatrics and the result was in line with previous IVPD and IVPG measurements in dogs [64], suggesting the possibility of CMME-derived IVPD and IVPG to be examined in dog breeds at high risk of cardiac anomalies.

In addition, CMME-measured variables were evaluated in a clinical study of dogs with PDA [63]. PDA is an excellent illustration of volume overloading since it causes a severe left-to-right shunt from the aorta to the pulmonary artery, which results in left-side dilatation, the elevation of end-diastolic pressure, cardiac failure, and overt pulmonary edema [65]. In our recent study, the short-term alterations in IVPD and IVPG in dogs before and after surgical occlusion of ductus arteriosus were demonstrated to identify the possibility of novel indices in the rapid detection of changes in LV relaxation [63]. The results revealed that CMME-derived IVPD and IVPG indices were able to reflect reduced preload after the ductus closure, as indicated by the reduction in total and basal IVPD and IVPG, but not the relaxation of the LV. According to the previous study, it was speculated that the changes in IVPD and IVPG, which occurred 48 h after the occlusion of ductus arteriosus, did not take place over a sufficient time to observe LV relaxation properties; this may require long-term observation [63]. In another study, mid-to-apical IVPG was improved in dogs after one-month post-ligation of the ductus arteriosus, suggesting improved suction force and relaxation of the LV (unpublished data).

D. ***IVPD and IVPG in pigs***

Cardiomyopathies, or conditions that affect the cardiac ability to pump blood, can be inherited or acquired; they can manifest as arrhythmogenic, dilated, hypertrophic, ischemic, or restricted conditions. Complex diagnostic standards must be used for these various types of heart failure, and both the pathophysiology and the approach to treatment vary greatly [66]. Dilated cardiomyopathy (DCM) is the third most common heart disease in humans and the second most common heart disease in dogs. In human studies of DCM, there are more than 50 genetic loci associated with the disease, and canine DCM has a similar disease progression to that of humans [67].

The right ventricular (RV) IVPD measured by CMME in a pig model with changed preload, afterload, and lusitropic states as well as in human patients with DCM has been studied [68]. In pigs, the RV pressure difference was created by inertial forces which were correlated with the RV diastolic force measured by micromanometers. In human patients with DCM, the right ventricular IVPD was significantly reduced, reflecting the severity of the impaired RV relaxation, which can be used for the assessment of RV diastolic function. In some dog breeds, DCM is frequently linked to genetic susceptibility. It can also develop secondarily as a result of concurrent illnesses and dietary deficits [69]. Some studies demonstrated the efficiency of STE for the early detection of contraction abnormalities in dogs with DCM [70,71]. In addition to the systolic failure in dogs with DCM, moderate and severe DD are observed in occult and overt DCM [72,73]. Thus, the combination of new SET, IVPG, diet, and established inheritance patterns of DCM in some dog breeds which are at high risk of DCM is worth clinical investigation.

E. ***CMME-derived IVPD and IVPG in sheep and goats***

Small ruminants, especially goats and sheep, serve as good experimental models for cardiovascular research purposes because of the average body size, easy management, and relative heart size to those of humans and companion animals [74,75]. In addition, they serve as a potential model to discuss the pathophysiological changes in large ruminants when complex experimental techniques cannot be performed in large animals under farm conditions [76,77].

CMME has been studied in sheep and goats. In the sheep model of the LV aneurysm [78], simultaneous catheterization and CMME were performed, and the results revealed a good correlation between IVPG obtained from CMME and catheterization. Normal sheep exhibit linear CMME of mitral inflow, while aneurysm sheep showed an abrupt reduction in the inflow propagation velocity.

Recently, the feasibility of CMME-derived IVPD and IVPG measurements in wakeful and sedated goats as well as after pharmacological modulation of the heart was evaluated. Assessment of IVPD and IVPG from CMME was possible in goats and can be used for further cardiovascular experimentations [79]. In goats, as in dogs, both IVPD and IVPG were expressed as total, basal, mid-to-apical, mid, and apical parts. The IVPD and IVPG data in normal goats were comparatively similar to the obtained data from canine studies. Upon examination of goats with CMME, the effect of sedation with xylazine on CMME variables, including the total, basal, and mid-parts of both IVPD and IVPG, was significantly reduced in sedated goats compared with the baseline [79]. A recent study revealed that acute administration of melatonin, a pineal hormone with antioxidant, cardiovascular, and reproductive functions, enhanced testicular hemodynamics and metabolomics in goats [80]. We observed that intravenous melatonin administration ameliorates IVPG variables in goats without a significant effect on systolic function [81].

## 5. Discussion and Future Perspectives

The clinical relevance of IVPD and IVPG measured by CMME as promising methods for the early diagnosis of cardiac dysfunction is still under research consideration in laboratory animals, pets, and humans for future validation in clinical cardiology. Characterization of the intracardiac flow dynamics via IVPD and IVPG assessment in feline and canine pediatric cardiology may be important to deepen our understanding of the pathophysiology, prognosis, and follow-up treatment courses, especially when the continuous assessment of the diastolic function is needed.

The physiologic variation of IVPD and IVPG in client-owned healthy cats has been established [25]. Future studies will open the door to further investigation of IVPD and IVPG in cats with HCM, which is a good example of impaired myocardial relaxation.

Concerning congenital heart disease (CHD), the evaluation of myocardial function has been widely researched. The systolic function has received the most attention in CHD to date due to its significance and measurement simplicity. However, the diastolic function as an important component of LV function is often overlooked. The conventional parameters which are used to assess diastolic function often measure the blood flow and are affected by the loading conditions of the heart. The problem in diastolic function assessment occurs when attempts are made to evaluate the myocardial relaxation independently of the preload and/or afterload. The interpretation of the diastolic function in the context of CHD requires some understanding of the effects of the lesions themselves on the established diastolic function parameters [82]. Careful and exhaustive analysis of non-invasive IVPD and IVPG via CMME away from catheterization may help in precise diastolic function evaluation, explanation of symptoms, and better follow-up in individuals with preserved ventricular function.

Another imaging technique, STE, can accurately and quantitatively evaluate diastolic and systolic myocardial performance [70]. A detailed study uncovering the association between IVPD and IVPG and STE indices, particularly in CHD, will be valuable. The notion has been partially covered in the previous studies by Matsuura et al. in canine models of loading changes, chemotherapy-induced heart failure, and healthy cats [25,26,56].

Chemotherapeutic agents are known to adversely affect heart function either in an early or latent manner. Few studies demonstrated the efficiency of IVPD and IVPG in chemotherapy-related heart failure in humans and dogs [26,62]. Since changes in the intraventricular blood flow dynamics occur earlier than the wall changes, intensive studies are needed to examine the effectiveness of these novel indices in the early detection of heart failure after chemotherapy. In addition, the relationship between CMME variables and cardiac biomarkers has not been investigated, which requires studying animal models and clinical investigations.

The current trend in cardiovascular disease diagnostic techniques is aimed at the prediction and early diagnosis of heart failure before the onset of clinical symptoms. For the efficient use of various animals as a candidate for cardiology research, novel echocardiographic techniques are also pertinent for validation in the used model for the accurate interpretation of pathological or pharmacological conditions [79,83,84]. Consequently, it is important to continue researching the physiology of IVF in various animal species, including farm animals, particularly in species in which a well-aligned apical view is achievable, such as small ruminants. In this regard, serial studies have been conducted on goats; however, only one study has been published [79]. Assessment of IVF in some farm animals, such as goats, might explore new aspects of the pathophysiological consequences of cardiometabolic disorders concerning pharmacology, age, level of nutrition, and production status [76,77,79,85,86,87,88]. Concomitantly, this also will provide a step forward in the clinical validation of these methods in humans and animals. Deaths and severe economic losses due to heart problems are frequently occurring in ruminant species; however, diagnostic facilities for early detection and accurate diagnosis of cardiac diseases in ruminants are still lacking [86,87]. Moreover, small ruminants such as sheep and goats are useful animal models to study the pathophysiology of ruminants and at the same time are considered efficient models for cardiovascular research [88,89,90].

Thus, the CMME-derived IVPD and IVPG have important theoretical significance and a wide range of application prospects in cardiac disorders as new imaging indices, particularly in the evaluation of diastolic function. In moving away from catheterization, IVPD and IVPG from CMME could be new echocardiographic indices that reflect the diastolic function changes and, in the future, may replace catheterization procedures to diagnose changes in diastolic function. In veterinary medicine, many research trials should be concerned with upcoming studies.

## Figures and Tables

**Table 2:** Color M-mode-derived intraventricular pressure indices in canine experimental models.

Experimental Model	Chemotherapy CM		Overloading		
Time	After 4.5 Months	After 18 Months	Pressure Load	Volume Load	Milrinone
Total IVPD	ND	ND	↓	↑	NS
Basal IVPD	ND	ND	NS	NS	NS
Mid-to-apical IVPD	ND	ND	NS	ND	ND
Mid IVPD	ND	ND	↓	NS	↑
Apical IVPD	ND	ND	NS	↑	NS
Total IVPG	NS	↓	ND	ND	ND
Basal IVPG	NS	NS	ND	ND	ND
Mid-to-apical IVPG	NS	↓	ND	ND	ND
Mid IVPG	↓	↓	ND	ND	ND
Apical IVPG	NS	NS	ND	ND	ND
References	[26]		[56]		

CM, cardiomyopathy; ND, not determined; NS, not significant. Arrows are used to indicate an increase or decrease.

## Data Availability

The collected literature is available on request.
